# Training the algorithm: Agency and algorithmic injustice in Instagram’s ‘whitewashed’ health and fitness spaces

**DOI:** 10.1177/14614448251366172

**Published:** 2025-09-24

**Authors:** Hester Hockin-Boyers, Patricia Vertinsky, Moss Norman, Nikolaus A. Dean, Aishwarya Ramachandran

**Affiliations:** Durham University, UK; The University of British Columbia, Canada; The University of British Columbia, Canada; University of Waikato, New Zealand; The University of British Columbia, Canada

**Keywords:** Agency, algorithms, body image, content elicitation, fitness culture, Instagram, race, social media

## Abstract

Research on women’s body image has often focused on the potential harms associated with engaging with health and fitness content on Instagram. Recently, scholars have turned their attention to exploring women’s agency in relation to social media to examine how particular groups of individuals participate in the curation of their online worlds in pursuit of a positive body image. In particular, we consider the experiences of specific racial groups who are interacting with what some claim are ‘whitewashed’ and algorithmically biased digital environments. We aim to contribute to this enquiry by drawing on semi-structured interviews and ‘content elicitation’ with 32 Chinese Canadian women who were invited to describe their experiences with health and fitness content on Instagram. We found that our participants displayed a heightened awareness of algorithmic bias and in response, attempted to actively ‘train’ their algorithms to provide content that reflected greater bodily diversity.

## Introduction

Much of the current research on body image and social media is underpinned by a logic of ‘risk’ and ‘exposure’, focusing on the internal mechanisms through which the consumption of certain media may impact body image and well-being (Danthinne et al., 2022; Fioravanti et al., 2022; Sanzari et al., 2023). Scholars have also begun to explore the ways in which social media users actively curate their social media worlds by ‘unfollowing’, muting and blocking content that they feel contributes to a negative body image, as well as actively seeking out content that supports a more positive body image^1^ (Hockin-Boyers et al., 2021; Greene et al., 2022; Mahoney, 2022; Tylka et al., 2023). These studies aim to move beyond simply examining the perceived harms of consuming certain content, instead advancing a more nuanced framework that better accounts for women’s agency in their social media use and its relationship to body image. In short, this research asks; rather than passively absorbing social media content, how do women actively and critically engage with what they encounter online- and how does this, in turn, shape their everyday behaviours on social media, such as scrolling past, (un)following, ‘liking’ or intentionally searching for content? Given the participatory benefits social media platforms may provide in contrast to ‘traditional’ media forms (Jenkins et al., 2016), a greater focus on agency may be especially helpful in understanding body image experiences in selected contemporary media landscapes.

While research on body image in social media increasingly recognizes the role of agency in shaping users’ experiences, there remains an opportunity to explore how minoritized identities may shape women’s experiences online in specific ways, particularly how they understand and interact with social media algorithms. Although the number of studies at the intersection of body image, race, and social media use are increasing (DeBraganza and Hausenblas, 2010; Opara and Santos, 2019; Ward et al., 2023), much of the existing research has primarily focused on the experiences of white, college-aged female subjects (Harriger et al., 2023; Perloff, 2014; Rodgers et al., 2022). This overemphasis on one particular type of experience is largely due to the frequent use of convenience sampling among college populations. Harriger et al. (2023) note, for example, that minority racial and ethnic groups, such as East Asian women, are chronically underrepresented in studies of body image. Exploring how gender and race intersect as women actively navigate social media environments is thus important, since digital scholars exploring racial issues have consistently pointed to the ways in which racial biases and stereotypes become embedded and amplified through social media algorithms (Benjamin, 2019; Bishop, 2025; Epps-Darling, 2020; Noble, 2018). In this sense, the experiences of racially minoritized groups,^2^ who may be interacting with ‘whitewashed’ and algorithmically biased digital environments, may provide new insights to the body image field.^3^

To address this gap, our study examines the social media experiences of Chinese Canadian women, who have often been described as a ‘visible minority’ in Canada (Jette et al., 2014). According to current census data, Chinese Canadians make up 4.7% of the Canadian population, with substantially higher population rates in certain municipalities (Statistics Canada, 2024). In Canada, the Chinese population is a linguistically and culturally diverse group that consists of individuals with an array of backgrounds and experiences, ranging from recent immigrants to those whose families have contributed to the fabric of Canada for generations. Despite their significance as a diaspora group and relative visibility in Canadian society, little is known about how Chinese Canadians are represented on Western social media platforms like Instagram. Notably, these platforms are not governed by principles of proportional representation, but by algorithmic systems shaped by popularity metrics, commercial interests and user engagement patterns. This makes it especially important to explore how Chinese Canadian women experience and interpret algorithmic visibility, particularly in relation to representation and body image, as their voices are currently underrepresented in the existing body image literature (Harriger et al., 2023).

With regard to the type of content through which we explore body image and algorithmic injustice, our focus is on health and fitness content on Instagram. We define this broadly as posts, stories, or reels that promote physical activity, nutritional practices, body-related aesthetics or wellness routines. This genre has attracted sustained scholarly attention in body image research, particularly due to the popularity of ‘fitspiration’^4^ content and its frequently reported negative effects on body image, self-esteem and mood (Drummond and Tiggemann, 2017; Jerónimo and Carraça, 2022; Slater et al., 2017). Characterized by highly aesthetic and normative presentations of gendered body ideals, most notably the ‘slim-thick’^5^ feminine aesthetic (McComb and Mills, 2022), health and fitness content offer fertile ground for investigating how minoritized women engage with and negotiate body image and belonging in digital environments. This content focus also aligns with the interdisciplinary orientation of the research team, whose expertise spans kinesiology, body studies and the sociohistorical analysis of physical cultures.

Since body image is a complex construct that is grounded in a person’s broader identity (Gahler et al., 2023; Keigan et al., 2024), it is helpful to consider how racialized women make sense of body image and body belonging in digital health and fitness spaces, especially where they may not see their own identities reflected in a way that appeals to them. Moreover, research has yet to explore whether and to what extent women deliberately ‘seek out’ health and fitness practices that may align with their racial and ethnic identity and to what extent this is a conscious practice. By engaging with a sample of Chinese Canadian women, an underrepresented population in body image studies, we sought to gain new insights concerning women’s agency in shaping their social worlds. In the following sections, we first explore media research on the inequalities embedded in social media algorithms and users’ interpretations of these systems. We then shift focus to user agency, examining literature on the productive and curatorial strategies that social media users employ.

### Algorithms and inequalities

Algorithms can be viewed as digital tools used to ‘answer (and enact) the question, what should be made visible, and to whom?’ (Bishop, 2019: 2590). In the context of health and fitness content on Instagram, this translates to decisions about which bodies and health practices are promoted or prioritized for visibility among users. Algorithms are a defining aspect of social media architecture, yet making any sort of truth claim about algorithms is difficult, given that the technical logics of these decision-making entities are not in the public domain (Andersen, 2020; Bishop, 2019). Algorithms are typically referred to as ‘black boxes’ that can be analysed only in terms of their inputs and outputs (Ananny and Crawford, 2018; Christin, 2020). As a result, social researchers tend to look to the accounts of everyday users to explore how algorithmic processes are experienced and theorized, rather than focusing upon the technical attributes of algorithms (Bishop, 2019; Bucher, 2017; Schellewald, 2022).

Variously called ‘algorithmic imaginaries’ (Bucher, 2017), ‘algorithmic gossip’ (Bishop, 2019), ‘folk theories of algorithms’ (Ytre-Arne and Moe, 2021) and ‘stories of algorithms’ (Schellewald, 2022), this arena of scholarship emphasizes the notion of ‘algorithms as culture’, which seeks to define algorithms ‘not through their inside, so to speak, but rather along their outside edges, the ways in which they are integrated into society and the lives of individuals encountering them’ (Schellewald, 2022: 2). In this sense, while algorithms are designed to have a naturalistic, even inevitable feeling, what Boulton and Zook (2013) refer to as ‘the duplicity of code’ (p. 440), they have subtle yet perceptible impacts on the various experiences of social media, which users are able to reflect upon, create stories about and strategize around. Low et al. (2025) contend that the algorithm’s dynamicity is ‘part of what makes them invisible – or rather, forgettable – for most users. It is mainly when something is amiss that users notice the presence of algorithms’ (p. 2339). What remains relatively underexplored, however, is whether this sense of something being ‘amiss’ – the moment when the algorithm becomes visible – varies depending on a user’s identity and their perceived visibility within these digital spaces.

Identifying how algorithms sift through and ‘throw up’ certain images and bodies over others is clearly essential to understanding minoritized women’s experiences of health and fitness content on Instagram, and its impact on body image. As research by digital scholars demonstrates, inequalities and racial biases are amplified by algorithms, which have been observed to privilege discourses that reproduce harmful stereotypes or inaccurate information (Benjamin, 2019; Epps-Darling, 2020; Noble, 2018; Riccio et al., 2024). For example, in her article on how artists strategically navigate the ‘platformisation’ of art worlds, Bishop (2025) shared how one artist noticed that her portraiture of light skinned Black women was more visible and favoured by the algorithm over her portraiture of dark skinned women. Bishop (2025) argues that algorithmic systems ‘facilitate the discrimination of women of colour through automated forms of (mis)identification and overzealous moderation that, together, reduces their visibility’ (p. 2114). This underscores the importance of interrogating how algorithmic processes shape what types of bodies are surfaced or suppressed, particularly for racialized women. In the context of our study, this framing helps situate Chinese Canadian women’s experiences within broader conversations about digital (in)visibility, algorithmic bias and the everyday negotiations required to claim space in aestheticized, racially coded fitness environments online.

It has also been argued that algorithms seek to reinforce white, male, heteronormative and neurotypical ways of engaging socially (Bishop, 2021; Goodman, 2020). Hence, the racially biased nature of algorithms is symbiotic with wider societal prejudices in that it informs and is informed by existing ‘naturally occurring’ racial bias (Benjamin, 2019). In this sense, as users’ experiences become entangled with algorithms (Gillespie, 2014), existing racial prejudices can be learned by platforms – a logic which, over time, becomes embedded, reproduced and spread.

The commercial nature of these digital platforms, which profit from promoting content that engages users and encourages them to generate more clicks and views, lies at the heart of perpetuating such inequalities (Noble, 2018). As Van Dijck (2013) notes, ‘popularity is not simply out there, ready to be measured: it is, rather, engineered through algorithms that prompt users to rank things, ideas, or people in relation to other things, ideas, or people’ (p. 62). In this sense, social media’s ‘attention economy’ is highly relevant to discussions of body image on social media (Van Dijck, 2013), where white, thin, able-bodied and heteronormative body ideals may be coded as potentially more ‘commercially successful’ or ‘popular’ on platforms like Instagram and are therefore promoted by the algorithm over non-conforming or racially othered bodies^6^ (Carah and Dobson, 2016; Williams, 2021). The privileging of whiteness in this context aligns with what Epps-Darling (2020) describes as ‘technological microaggressions’, subtle, algorithmically driven forms of exclusion that reinforce systemic inequalities through platform design. Although there has been important work in the body image field that points to the significance of platform algorithms in shaping social media users’ experiences of body image and body belonging (Griffiths et al., 2024; Gurtala and Fardouly, 2023; Harriger et al., 2023; Joiner et al., 2023), we contend that more attention is needed to locate these experiences within the broader, commercially driven and algorithmically curated media landscape.

### Algorithms and agency

Research on women’s interactions with health and fitness content on social media has traditionally been dominated by psychological frameworks that tend to measure body image as a response to media exposure (Danthinne et al., 2022; Fioravanti et al., 2022; Sanzari et al., 2023). As Rich (2024) observes, ‘body disaffection is often framed as the “effect” of a causal relationship between simply viewing an image and the development of low self-esteem’ (p. 702). However, recent media scholarship has aimed to add nuance to these perspectives, stressing the importance of understanding how women engage with social media platforms and their algorithms in complex and agentic ways (Coffey, 2021; Rich, 2024). In what follows, we examine the literature on these agentic approaches and consider its constraints.

Social media is a participatory medium (Jenkins et al., 2016). When women encounter health and fitness content on these platforms, they have a broad range of possible options for engagement. These include what Picone et al. (2019) describe as ‘small acts of engagement’ – such as liking, sharing or commenting, and what Rose (2016) refers to as ‘friction’, the subtle, sometimes resistant interactions users have with digital content. For example, users can like, resist, unfollow, share, scroll past or mute this content, depending on their interpretations of its meaning or reflections on its possible impact on well-being following repeated exposure. As agentic beings, social media users can strategize around algorithms to shape their own online environments (Adams et al., 2020; Bishop, 2019; Kapsch, 2022). In this regard, social media users do more with algorithms than create stories or share theories about their inner workings; they also engage with them in productive ways to achieve specific ends.

Of particular relevance to this study is the extensive body of literature examining digital curation practices (Hockin-Boyers and Clifford-Astbury, 2021; Hockin-Boyers et al., 2021; Davis, 2017; Fotopoulou and Couldry, 2015), which can be conceptualized as the practice of selecting ‘that which is relevant, ignore, or marginalize that which is less so, and make such decisions ‒ though often subconsciously ‒ based upon definitions of the situation, and definitions of the stimuli themselves’ (Davis, 2017: 771). Such engagement entails a dynamic interplay between user agency and algorithmic systems, with users actively shaping, organizing and managing digital content in ways that reflect their values, aspirations, and desires. Practices like hash-tagging, blocking, muting, selecting and sharing posts, and cultivating aesthetic cohesion within social media feeds function as curatorial acts that can grant users a sense of ownership and control over their digital environments (Merten, 2021). These acts also reveal how digital curation is both a means of personal expression and a navigation of platform logic designed to prioritize certain forms of visibility and interaction.

One of the more comprehensively theorized forms of agentic engagement with algorithms is found in the field of influencer^7^ studies, which conceptualizes this engagement as a form of ‘digital labour’ (Bishop, 2021; Ferrari and Graham, 2021). For influencers, maintaining visibility and consistent engagement is crucial for their financial success and professional viability. As a result, understanding how to ‘game’ or strategically interact with algorithms becomes an ongoing project requiring a blend of diligence, adaptability, and technological savvy (Cotter, 2019). This form of labour often involves monitoring changes in platform algorithms and adapting content strategies to align with shifting priorities. Davis (2017) refers to this kind of curation as ‘productive curation’, concerned with the presentation of the self and visibility management. However, as this study primarily explores how Chinese Canadian women navigate content produced by others, we are more interested in ‘consumptive curation’; a practice through which ‘networked individuals navigate pools of data in discriminating ways’ (Davis, 2017: 773).

Yet, as feminist materialist scholars argue, user agency is complicated by the notion that platforms themselves are productive and somewhat agentic entities, determining how users engage (through their affordances), and nudging them to consume and behave in various ways (Coffey, 2021; Rich, 2024). As Van Dijck (2013) aptly contends, ‘a platform is not a thing; it makes things happen’ (p. 180). In this regard, algorithms seek to not only predict user preferences, but shape them. Lazer et al. (2024) call this process ‘agency hacking’, whereby information or content may become ‘appealing in such a way that it undermines priorities set by the individual’ (p. 749). Thus, while users do possess some degree of agency in relation to social media, their agency is somewhat constrained by wider factors such as the platforms’ affordances and architectures, including, but not limited to, its algorithm. This article adds to the growing body of research on social media users’ interactions with algorithms by exploring how minoritized women perceive health and fitness content on Instagram and navigate their own sense of agency within these algorithmic structures.

## Methods

This project piloted a new qualitative method, coined by the authors as ‘content elicitation’. The method follows the photo-elicitation tradition by asking participants to collect visual artefacts that might stimulate discussion and establish shared meaning (Hackshaw-McGeagh et al., 2018). However, instead of photos ‘found’ or taken on cameras, content elicitation in this study asks for insight into participants’ everyday social media feeds using a taken-for-granted feature of the app; direct messenger. Participants were asked to send health and fitness content^8^ they encountered in their everyday Instagram use and direct message any content that engendered a positive or negative reaction to the lead researchers’ alias Instagram account. At the end of the week, the lead author conducted semi-structured interviews over Zoom with each participant to discuss their engagements with health and fitness content on Instagram and to go through and discuss the content they had sent over the previous week.

Our use of content elicitation in this study draws on methodologies such as social media ‘walk-through’ approaches (Light et al., 2018) and visual curation techniques (Persohn, 2021), where participants are invited to actively guide the research process through a ‘show and tell’ format. This method foregrounds participants’ own insights and experiences, facilitating an interactive, participant-driven exploration of their digital environments. We found this approach particularly valuable for capturing the unique, subjectively curated ‘corners’ of the Internet that participants inhabit. Whereas relying on pre-existing labels to define health and fitness content (e.g. ‘fitspiration’) risks flattening the diversity of content experiences on social media, content elicitation placed the emphasis on participants themselves to define what constituted health and fitness content, including its boundaries, intersections and points of overlap with adjacent categories such as wellness, mental health and self-improvement.

It is noteworthy that while this method was operationalized to explore participants’ social media ‘worlds’ in the context of health and fitness, our approach also has utility in exploring any number of digital content types. Furthermore, although we piloted the method on Instagram due to its existing affordances (the direct messenger feature and algorithmically generated ‘feed’), it could also be used ‘cross-platform’. For example, one might use the messenger feature of TikTok, X or the ‘pin’ feature of Pinterest to create a collection of content on a specific theme.

While this method is virtual in the sense that it is a digitization of a traditional research method (photo-elicitation) (Venturini et al., 2018), we also suggest that by virtue of the creative re-purposing of existing platform affordances (Instagram’s feed, algorithm and direct messenger feature), it is more usefully categorized as ‘natively digital’ (Rogers, 2013). The ‘DM’ (direct message) is a commonly used social feature of the platform, allowing users to share content and converse with friends. Operationalizing this feature for research purposes enables us to effectively ‘follow the medium’ in a way that feels natural and is complementary to the user’s everyday social media use (Rogers, 2013), thus reducing the time/effort burden of data collection for participants.

In practical terms, health and fitness content were sent over the course of a week via DM to the lead researcher’s alias Instagram account, which served as an effective ‘digital base’ for data collection. Collecting social media content via DM enabled timestamped data to be collected in one place (the lead researcher’s inbox); hence, during interviews, the lead researcher could refer back to this inbox to find and discuss the content shared. Given that the focus of this article was the participants’ strategic navigation of Instagram rather than the content itself, data from the content elicitation exercise (Instagram posts) have not been included in our findings.

Following a week of content elicitation, the lead researcher conducted semi-structured interviews in English^9^ with participants over Zoom, typically lasting 1–2 hours. Since data collection took place from April to May 2021, a time when Canadian provinces were in various phases of COVID-19 lockdown, all interviews were conducted online. In a way, conducting online interviews resonated with the project’s objectives, which were to explore the online worlds of a diasporic group. The research team, comprising members from various continents, also convened solely through digital platforms, rendering the project quintessentially digital in nature. A notable limitation was the occasional technical issue, such as poor sound quality or unstable Internet connections. One advantage of conducting online interviews was the ability to recruit participants from across Canada, including any territory or province, although most were from British Columbia and Ontario.

Content elicitation was used with interviews to create a sense of shared understanding about the highly personal and individualized nature of Chinese Canadian women’s engagement with health and fitness spaces on Instagram. Combining these methods enabled us to achieve a sense of the participants’ social media environments, as well as their personal reflections on how they navigate and interpret this digital space. At the start of the content elicitation exercise, participants were invited to share their reflections on the process, fostering open dialogue and prompting them to consider how they selected content and what thoughts or feelings motivated them to direct message the lead researcher.

Content elicitation was used alongside interviews to gain insight into how Chinese Canadian women navigate algorithmic bias and body ideals in Instagram’s health and fitness spaces. Rather than analysing the content participants shared in detail, the exercise served as a prompt for reflection, anchoring discussions in participants’ lived experiences of engaging with this highly individualized digital environment. The method created a shared entry point into their social media worlds, allowing participants to articulate how they interpret, manage and respond to the content shown to them by the platform. At the start of the content elicitation exercise, participants were invited to reflect on what informed their selection and what thoughts and feelings prompted them to direct message the content to the lead researcher, giving rise to insights into perceived algorithmic biases.

Interview transcripts and content elicitation data were then analysed using reflexive thematic analysis in order to identify patterns of meaning across the qualitative dataset (Braun and Clarke, 2019). Reflexive thematic analysis values researcher subjectivity and reflexivity, viewing coding as an interpretive, not objective, process where meaning is constructed rather than discovered. The lead author conducted the initial coding and led the development of themes. The broader author team contributed through regular discussions, where developing themes were reviewed, reflected upon and refined collaboratively to ensure interpretive depth and analytic rigour. Rather than unfolding as a rigid, step-by-step process, the analysis was iterative and dynamic, allowing for continuous movement between data, codes and themes. This dynamic approach enabled continual refinement and re-interpretation of themes, capturing the fluid and emergent nature of qualitative inquiry.

In the data analysis process, we employed an inductive approach, attending to the explicitly stated ideas, concepts and meanings expressed, as well as the less explicit latent ideas communicated through language, tone and context. For example, participants often described the algorithm as a non-human actor that could prompt a range of reactions and responses. The algorithms’ agency became apparent through the choice of verbs, for example, ‘the algorithm shows me . . .’ and ‘the algorithm notices I’m looking . . .’. This use of language reflects, in some ways, the participant’s relationship to the platform and their understanding of the algorithm’s role.

While the data presented in this article forms part of a wider project exploring Chinese Canadian women’s body belonging, engagement with physical activity and social media use, the themes we explored in this article (e.g., ‘algorithms’, ‘agency and social media’, ‘whitewashing of Instagram’), represent one thematic cluster resulting from the analysis of the data set. The thematic strands presented below, ‘notions of algorithmic injustice’ and ‘training the algorithm’, developed as recurrent conceptual ideas that tied women’s stories together and created a sense of clarity and coherence within the data presented.

### Ethics and privacy online

Collecting data via content elicitation required careful negotiation of privacy, both for the participant and for the Instagram users whose content was being shared with the researchers. With regard to participant privacy, the authors did not observe or record any data from the participants’ own Instagram profiles, other than their username, which was simply recorded for the purpose of distinguishing between direct message chats with various participants. This is because our research was interested in interactions with content produced by others, rather than content that participants produced themselves. Once both data collection phases (content elicitation and interview) were complete, the lead researcher securely recorded all relevant data from the direct message chats (images, captions and messages about the content) on their University OneDrive account. Another ethical concern was the privacy of Instagram users whose content was being shared and discussed without their knowledge. In order to disguise their identity, identifiable features of these Instagram users (e.g. usernames) were removed from the interview transcripts. The research also received institutional ethical approval from both collaborating Universities.

### Participants

Our participants included 32 first- and second-generation Chinese Canadian women, aged 19–37, who were currently living in Canada (see Table 1). Of these participants, 15 identified as first generation and 17 as second-generation Chinese Canadians. For consistency, our study adheres to Statistics Canada’s definition of generation status whereby first generation refers to people living in Canada who were born outside Canada and second generation refers to individuals who were born in Canada and had at least one parent born outside Canada (Statistics Canada, 2021).

**Table 1.** Participant information.

Pseudonym	Location	Generation status	Age	Occupation
Alexia	Vancouver (BC)	1st Gen	29	Real estate agent
Alice	Vancouver (BC)	2nd Gen	22	Student (Business)
Caroline	Toronto (ON)	2nd Gen	34	Flight attendant
Celeste	Vancouver (BC)	2nd Gen	31	Childcare worker
Charlie	Montreal (QC)	1st Gen	32	Data analyst
Claire	Kitchener (ON)	1st Gen	27	Music therapist
Elise	Toronto (ON)	2nd Gen	34	Consultant
Emma	Vancouver (BC)	2nd Gen	21	Student (Medicine)
Flora	Toronto (ON)	1st Gen	33	Teacher
Frances	Vancouver (BC)	1st Gen	31	Waitress
Francesca	Vancouver (BC)	1st Gen	32	Teacher
Gemma	Vancouver (BC)	1st Gen	31	Call centre worker
Imogen	Vancouver (BC)	2nd Gen	27	Finance
Jenna	Vancouver (BC)	2nd Gen	19	Student (Kinesiology)
Julia	Toronto (ON)	2nd Gen	32	Marketing
Justine	Toronto (ON)	2nd Gen	34	Family therapist
Kara	Vancouver (BC)	2nd Gen	30	Teacher
Lilly	Toronto (ON)	1st Gen	32	Architect
Lorayne	Gibsons (BC)	2nd Gen	31	Youth coordinator
Lorna	Vancouver (BC)	2nd Gen	25	Teacher
Mindy	Toronto (ON)	1st Gen	31	HR administrator
Miranda	Vancouver (BC)	1st Gen	36	Administrative assistant
Morna	Vancouver (BC)	2nd Gen	37	Stay at home mom
Rachel	Vancouver (BC)	1st Gen	23	Physical therapist
Rose	Vancouver (BC)	1st Gen	32	Interior designer
Sadie	Toronto (ON)	2nd Gen	31	Lawyer
Sarah	Toronto (ON)	1st Gen	33	Flight attendant
Sasha	Toronto (ON)	2nd Gen	29	Flight attendant
Serena	Toronto (ON	1st Gen	28	Marketing
Stacey	Vancouver (BC)	2nd Gen	22	Student (Kinesiology)
Sophie	Waterloo (ON)	2nd Gen	23	Data analyst
Victoria	Toronto (ON	1st Gen	34	Stay at home mom

Participants were recruited via snowball sampling (Noy, 2008). Initially, a recruitment notice advertising the project was posted to the lead researchers’ Instagram account and was shared by the project team among their networks. Following initial contact with two to three participants, the lead researcher asked participants to share the flyer with their networks, which snowballed over the course of 2 months to a total sample of 32 participants. Given the relatively time-intensive nature of data collection, participants were also offered monetary incentives ($35 gift vouchers) to take part. All participants were anonymized in the data and either chose or were randomly assigned a pseudonym.

**Table 2.** Inclusion and exclusion criteria for participant recruitment.

Inclusion criteria	Exclusion criteria
First- or second-generation Chinese Canadian	Third-generation or higher generation Chinese Canadian
Self-identifying woman	Identifies as a gender other than woman
Aged 18+	Under 18 years of age
Regular engagement with health and fitness content on Instagram	Does not regularly engage with health and fitness content on Instagram

The majority of participants lived in urban areas, which is partially reflective of the dispersal of Chinese diaspora, of which there is a higher proportion in metropolitan areas (Statistics Canada, 2024). This is also likely a consequence of using snowball sampling to recruit participants, as initial contacts in these urban centres were more likely to pass the study information on to friends and acquaintances who lived close by. This inevitably means the sample lacks a rural perspective, which is a limitation of the research.

Participants were all casual Instagram users who, semi-regularly, engaged with health and fitness content on the app. Participants generally had relatively small followings and typically privileged consuming content over creating their own. Through content elicitation and interviews, the lead researcher was able to develop an understanding of the types of health and fitness content participants engaged with on an everyday basis. Each participant’s social media ‘worlds’ differed greatly, with participants varying in their focus on specific forms of exercise or training styles (e.g. weightlifting, yoga, running) and health approaches, beliefs and lifestyles (e.g. intuitive exercise/eating, Traditional Chinese Medicine, body positivity, keto). Given the broad age range in the sample (19–37 years old), some differences in the types of content engaged with were observed, often reflecting participants’ life stages. Younger participants were more likely to share fitness content focused on physical appearance (e.g. workouts aimed at achieving a certain body type), while millennial participants more often engaged with content centred on ‘wellness’ and a more holistic approach to health and fitness (e.g. content on breathwork or correcting posture).

Given the project’s wider focus on Chinese Canadian women’s body belonging, engagement with physical activity, and social media use, interviews were explorative and broad in scope, covering a range of topics from exercise routines and online fitness culture to perceptions of racial discrimination and body image concerns. The data from this article thus focuses specifically on women’s experiences with health and fitness culture on Instagram and the content that participants found as helpful and harmful for managing their body image and well-being.

### Positionality and research context

While the research team comprises female and male researchers from different ethnic and cultural backgrounds, none share Chinese heritage with the participants in this study. It was therefore important that we reflected on our own positionality in relation to this topic, including reflexive thinking regarding our own biases and interpretations of the data (Carrington, 2008). For example, in a project of this nature, it was paramount to avoid essentializing East/West dichotomies and binary thinking (Said, 2003 [1978]). Accordingly, while we examined how women’s racial identity interacts with social media use and a sense of body belonging, we were mindful not to attribute every experience or data point solely to their Chinese heritage. Doing so could have an othering effect and risk overemphasizing racial identity at the expense of other equally important facets of participants’ lives and identities, such as their age, generational status or roles as mothers. Some members of the project team were more experienced in working with Chinese diaspora and Indigenous communities in Canada (Vertinsky et al., 2019; Norman et al., 2025); therefore, team meetings were invaluable for checking interpretations, acknowledging biases and ensuring analytical rigour.

With regard to the wider socio-political landscape within which this research is situated, data collection took place from April to May 2021, a period when Canadian provinces were in various phases of COVID-19 lockdown. At this time, there was also a resurgence of support for the ‘Stop Asian Hate movement’, due to reports of a number of attacks on Asians or Asian communities in North America (Cao et al., 2022). While this political movement was not at the forefront of data collection, we feel that it did play into the women’s accounts of how they experienced safety and body belonging on social media, which was a key site for the sharing of information and activism around these issues, as well as community, sociality and entertainment.

## Findings

### Notions of algorithmic injustice

Over Zoom, the lead researcher met with participants as they scrolled in unison through the direct messenger feed that participants had populated the previous week. Upon opening up their feed, it was common for participants to point out the homogeneity of the toned and ‘athleisure-clad’ bodies being observed and discussed. For Kara, sending health and fitness content for the project seemed to confirm previously held beliefs that her feed was what she called ‘whitewashed’. She pointed out that

I don’t know if you notice, but I only shared with you one post that featured people of colour, like my feed is so whitewashed, or just white in general, I should say, not whitewashed. But for some reason it is very uniform and homogenous in terms of the diversity that I see online that is fed to me. Being a Chinese Canadian, you don’t really see it [diversity] much.

Despite somewhat being architects of their social media environments, the Chinese Canadian women who took part in this study were clear that health and fitness spaces on Instagram lacked diversity, not only in terms of body type but also in terms of race. In this regard, participants perceptions of their Instagram feeds mirror social media research that argues algorithms disproportionately value white, thin, able-bodied and heteronormative bodies over non-conforming or racially othered bodies (Carah and Dobson, 2016; Noble, 2018; O’Connor, 2023; Riccio et al., 2024; Williams, 2021). For some women, like Kara, this observation was something they had often contemplated previously, and as a result, they were able to offer theories and observations about why and how their Instagram feeds had developed in this way. For others, scrolling through a week’s worth of content, collected as part of the content elicitation exercise, prompted new reflections on the kinds of body types they were routinely engaging with online.

Importantly, for many participants, including Kara, the perceived privileging of whiteness in this space had direct consequences for experiences of body image and body belonging. When asked how the experience of a ‘whitewashed’ feed made her feel, Kara mused,

It really impacts me . . . less so in physical wellness, but more my understanding and scope and shape of what beauty is. I always grew up with magazines and online, you see just a bunch of makeup how-to’s, and it never really fit on my face and I never could understand why . . . So, maybe that also applies to the beauty standards of what your body should look like and dimensions and those standards as well.

The notion that seeing bodies that reflect one’s own ethnic or racial identity in the health and fitness space as both important and beneficial was shared by most participants. As Jenna and Emma contended,

Jenna: I don’t really see people of colour in terms of in the fitness community . . . it would be nice to see people that are similar to me.

Emma: On social media, you see a lot of under-representation, I guess, of the Asian community. You see typically, like, western people with really nice bodies, and then you know- as a Chinese [woman], I know that is not the same physique that I have, and that is just because I was not born into that same kind of body. I think it can be unmotivating because that is the beauty standard that we are trying to go for, and we know that is not something that’s realistic for us.

The concept of ‘racial homophily’ – the tendency for individuals to form social connections with others who share the same racial or ethnic background – has been identified by body image researchers as influencing the types of bodies women are drawn to on social media (Gahler et al., 2023). While this sentiment was relatively ubiquitous across the sample, there were differences between how first- and second-generation Chinese Canadian women’s bodies and content was perceived. For instance, Rachel emphasized the relatability gap between fitness influencers who migrated during adolescence and those who have spent most of their lives in North America:

I have noticed that there is rarely a time where I would find someone or a Chinese influencer. Someone who is – I wouldn’t even say first-gen, but someone who maybe comes to Canada in their late teens or whatever . . . I personally tend to try to follow more the first-gen, second-gen Asians, because their body images relate more to what I think of myself.

For many participants, a unique blend of cultural assimilation and adaptation to Western beauty standards was crucial in fostering affinity with fitness content. Second-generation Chinese Canadian women often reflect a blend of Chinese heritage and Westernized health and fitness ideals, making their content especially relatable for those navigating similar cultural intersections. Conversely, first-generation influencers who immigrate during their teens or later may retain more traditional Chinese ideals, emphasizing qualities like slenderness or other aesthetic values (Goel et al., 2021; Kimber et al., 2015), which felt less resonant with participants who had grown up predominantly in Canada.

While experiencing Instagram as a space lacking diversity resounded across the sample, there was ambivalence regarding the underlying causes. Above, Kara frames the health and fitness content she observes online as something that is ‘fed to’ her. This articulation implies the latent inner workings of Instagram’s algorithm, which sifts through and throws up certain content. Here, the algorithm is afforded agency in affecting the shape and contents of Kara’s social media environment. In this sense, Kara’s account suggests a more deterministic view of the kinds of bodies she encounters, shaped by the underpinning architectures of the platform.

Conversely, in acknowledging the lack of body diversity in fitness spaces, some participants drew on discourses of choice and user agency in their explanations (Hockin-Boyers et al., 2021). For example, during her interview, Justine reflected that social media could often have a negative impact on her body image and mood. When asked why this was, she responded,

We don’t see a lot in terms of Asian representation . . . but it has to be our own project in order to raise it up, or bring it up kind of thing.

Similarly, when reflecting on the Instagram content sent over the course of the week, Victoria noted,

I don’t really get a lot of diversity of posts in fitness because I don’t seek them out.

This framing of social media feeds as an environment that can be subjectively designed and curated to reflect the users’ own tastes and values has been noted by scholars elsewhere (Davis, 2017; Fotopoulou and Couldry, 2015; Mahoney, 2022; Tylka et al., 2023). Hockin-Boyers et al. (2021) call this act of curation ‘digital pruning’, a practice whereby users engage in a process of unfollowing unhelpful or triggering content in the interests of protecting their own views and well-being. Digital pruning is framed as a long-term process requiring diligence and consistent upkeep. In engaging with digital pruning practices, which centre values of personal autonomy and choice, women take up some responsibility for the effects of social media on their body image and mood.

Many of the women who took part in this project offered a similar framing. For example, Stephanie contended,

I think the beauty and the curse of social media is that you can see what you want to see. So, if you follow people who are your body type, your ethnicity, then yes. But if you don’t follow them, then you won’t see them and so, I guess that really just depends on what you choose to look at. I think in that sense, social media acts a little bit differently because we used to . . . we used to see media on TV where you can’t tailor what you want; it just shows up, like billboards, it just shows up, bus station posters, you just see it. But now that we can personalise all of our media, it’s just up to you what you want to look at and who you want to look at.

Participants’ experiences on Instagram were fraught with feelings of a ‘lack of fit’, brought about by the privileging of whiteness in health and fitness spaces on Instagram. However, in combating this sense of alienation, women focused upon choice and user agency contending that, while their feeds seemed to lack diversity, it was ultimately their responsibility to curate a digital environment that reflected a broader range of bodies, both in terms of race/ethnicity and body type. In what follows, we further unpack how Chinese Canadian women work through their ideas around body belonging and algorithmic bias in an attempt to resolve or alleviate feelings of alienation and underrepresentation on the platform.

### ‘Training’ the algorithm

While participants in the study tended to only implicitly identify the algorithm as privileging white bodies, they did frequently allude to their perceptions of its latent inner workings as it sifted through and fed them certain content. When it came to exercising their own agency in relation to the health and fitness content they consumed, they did seem to emphasize an uptake of personal responsibility, autonomy and choice. Yet, they did not act alone. In attempts to diversify their feeds, participants invoked Instagram’s algorithm as a co-conspirator (Lazer et al., 2024; Nguyen-Thu and Munn, 2024), prompting it to assist in creating an environment that reinforced their need to see a range of bodies, particularly those that look like their own.

After asking some introductory questions to break the ice, the following exchange unfolded with Sasha,

Interviewer: How did you find the content elicitation exercise?

Sasha: I found that a lot of my fitness content is starting to come on the discovery page with Asian women.

Interviewer: Why do you think that is?

Sasha: I think that also comes to . . . there are not a lot of Asians in the wellness or the fitness industry . . . There are not a lot of Asian women influencers as much. So, I really seek them out and hope Instagram notices that I’m looking.

Like many other participants, Sasha pointed to a perceived lack of Asian representation in the health and fitness space on Instagram. As a solution to this, Sasha actively searched for women who share her East Asian heritage and hoped that Instagram ‘notices her looking’. The presence of Asian women on her ‘discovery page’,^10^ signifies that the algorithm has picked up her cues and is feeding her more content creators with Asian heritage. Here, the algorithm emerges as more than a latent and somewhat naturalized feature of the platform. Instead, it has become an active collaborator in the co-construction of women’s digital worlds. This collaborative relationship with algorithms has been observed elsewhere in digital research. For example, Nguyen-Thu and Munn (2024) explore how GrabBike workers in Hanoi strategize around algorithms in attempts to ‘tame’ the apps used to perform their work. In this research, learning ‘how the app thinks’ is an essential aspect GrabBike workers’ labour (Nguyen-Thu and Munn, 2024: 7). Conversely, in the present study, participants’ attempts to diversify and curate their Instagram feeds reflects a differently situated gendered and racialized form of labour (see also Mitra and Witherspoon, 2022).

One can see this through the experiences of Fran, who had started a ‘fitness journey’ during the first COVID-19 lockdown and was using Instagram to find exercise tips and motivation. Here, Fran displayed a similar spirit of collusion:

Sometimes I’m cognisant of- everybody I’m following, is everyone I’m following Caucasian? So, I try to have diversity in the people I follow, and someone told me about the algorithm that Instagram focuses more on Caucasians, and I wondered then, and I followed more people of diversity and I wondered about pop-ups on my feed.

Unlike Sasha, Fran has explicitly identified the algorithm as an entity she is trying to communicate with and to some extent, ‘correct’. In identifying the algorithm as the possible cause of a perceived lack of diversity she encounters in the health and fitness space, she experimentally attempts to ‘train’ the algorithm to reflect greater bodily diversity, particularly East Asian representation.

It is also noteworthy that ‘someone told’ Fran about the algorithm’s propensity to ‘focus more on Caucasians’. This reflects Bishop’s (2019) commentary on ‘algorithmic gossip’ whereby social media users share their theories and suspicions on the algorithm’s inner workings, as well as strategies to best harness its internal logics. As this work typically focuses on influencers and other platform workers, the findings in our study show that casual and everyday users engage in similar discussions, albeit towards different ends. In this sense, while platform workers often seek visibility and engagement, the participants in this study are motivated by a sense of personal well-being and a wish for representation.

While participants primarily used this algorithmic collaboration as a tactic to elicit a more diverse range of bodies on their feed, there were a number of other uses that also can be seen to fall within the category of countering ‘whitewashing’ in the fitness space. According to Charlie,

I do feel like I’ve got it [Instagram] tailored to what I want now. The only thing I would say is that . . . I would say we eat a mixture of Chinese food and- I say ‘white people food’, but that is just like a mix of Chinese and western diet. I am always searching for Chinese recipes, lots of tofu, stir fry and whatnot. But Instagram still just feeds me salmon and quinoa and it just doesn’t get that I eat other things!

While Charlie reported that she had mostly been able to ‘tailor’ her feed, the kinds of food cultures ‘fed to her’ can be viewed as staying in line with normative western middle-class constructions of ‘health’. Despite upholding her side of the collaboration by ‘searching’ for Chinese recipes, and thus signalling to the algorithm that her diet is varied and includes multiple food cultures, Instagram ‘doesn’t get’ the message being communicated. Thus, these findings represent a failure of the algorithm to correctly predict or influence the desires of racially minoritized users (Lazer et al., 2024). While participants felt they could somewhat ‘train’ the algorithm to reflect their preferred content, this curation had limits. Algorithms are complex, evolving ‘black boxes’ (Cotter and Reisdorf, 2020), and deeply embedded racial biases in the platform may be beyond the control of individual users (Lee, 2018).

## Concluding remarks

In our article, we explored how Chinese Canadian women experience body image and body belonging as they navigate health and fitness content on Instagram. Under the thematic heading ‘notions of algorithmic injustice’, our participants noted the predominance of white, slim and toned bodies on their feeds and shared their theories for this lack of diversity on the platform. While a number of the participants attributed this to Instagram’s algorithm, others emphasized the responsibility of Instagram users to seek out this content and collaborate with the algorithm in an effort to see a more diverse range of bodies on their feed, what we call ‘training the algorithm’. However, there remained some ambivalence regarding how effective this attempted collaboration was since, despite efforts to diversify content, some participants reported continued exposure to normative westernized ideals of health and fitness.

We suggest that these findings offer several valuable empirical insights. First, it is significant, although perhaps not surprising, that Chinese Canadian women perceive health and fitness content on Instagram as ‘whitewashed’ and lacking diversity. While algorithmic bias is now well established within digital scholarship (Benjamin, 2019; Noble, 2018; Williams, 2021), less research has explored the perception of everyday users as they navigate these embedded biases (for notable exceptions, see Armstrong, 2022; Turner and Hui, 2023). Second, it is interesting that when faced with this perceived lack of diversity, Chinese Canadian women actively sought out Asian creators to diversify their feeds and foster more positive body images. These findings echo the recent work of Gahler et al. (2023) who found that, for Asian-American women, following a higher percentage of racially similar accounts was positively associated with body appreciation and negatively associated with body dissatisfaction. Future research might usefully explore whether this agentic pursuit of diversity online is present in other minority populations in similar or different ways.

Another key finding from this study, which may have utility in social media research, is that the ‘natural’ and somewhat ‘inevitable’ feeling the algorithm is intended to induce is not necessarily experienced in the same ways by all social groups. Indeed, for minoritized groups, like the women in this study, the biases that shape users’ social media worlds are far from opaque (Low et al., 2025). Rather, the algorithms’ internal logics are perceptible in everyday interactions with platforms. Admittedly, since algorithms are shrouded in speculation and uncertainty, we cannot say these biases exist in absolute terms. In this sense, this article joins the growing line of work offering up ‘gossip’ and ‘folk theories’ into their inner workings (Bishop, 2019; Ytre-Arne and Moe, 2021), shaped by the lived experiences of users.

Our use of ‘content elicitation’ – a new method for exploring the social ‘worlds’ of participants – allowed us to capture a more agentic picture of social media, where content is liked, resisted, unfollowed, scrolled past and/or muted. In many ways, this emphasis on agency is intended as a corrective measure, devised to counter the passivity and ‘exposure’-driven narratives often seen in the body image field. This does not mean that we uncritically accept women’s agency in digital spaces and view it as a necessarily positive or empowering experience. On the contrary, our findings highlight the sense of responsibility Chinese Canadian women feel for seeking out and amplifying diversity on Instagram in focusing on their well-being. This is a kind of racialized digital labour that is less accounted for in social media research. In this sense, while our study shows that women can and do attempt to ‘train’ algorithms, this does not mean that they *should* or that such endeavours should not be troubled. Instead, our intention here is to demonstrate the various ways in which social media plays a role in reproducing and intensifying existing inequalities in relation to body image and body belonging.

## Notes

1. We follow Tylka and Wood-Barcalow’s (2015) definition of ‘positive body image’, which understands positive body image as a multifaceted, holistic construct that is distinct from negative body image, characterized by stability and flexibility that can protect well-being, and shaped by body acceptance from others and social identities.

2. In this research, we are interested in racial groups that may feel minoritized on Instagram specifically. Chinese Canadian women are likely to have a different experience on Chinese social media platforms such as Redbook (Xiaohongshu), where they are likely to be the majority racial group. We also use the term racially minoiritized as opposed to racial or visible minority to highlight the power relations embedded within constructions of race and processes of racialization. Although used by Statistics Canada, the language of ‘minority’ gives the impression that the marginalization racialized groups in Canada experience is merely a matter of numbers (i.e. their minority status) (Szto, 2020).

3. Throughout this article we use the nomenclature ‘body image’ which, being a psychological concept, somewhat conflicts with the articles’ more sociological underpinnings in body studies research. We have opted to use the language of ‘body image’ throughout as we intend for this article to have utility in the more psychology-oriented body image field.

4. Fitspiration content is defined as ‘images and messages that purport to motivate people to exercise and pursue a healthier lifestyle’ (Slater et al., 2017: 88).

5. The ‘slim-thick’ aesthetic is characterized by a narrow waist and flat stomach, with a fuller and more curvaceous butt, breasts, and thighs (McComb and Mills, 2022). While this body aesthetic is not ‘new’ in Black and Latino communities, its mainstream popularity in white-centred media is growing rapidly.

6. It is noteworthy to add that how and to what extent racial othering takes place is somewhat dependent on the platform itself and the geographical setting in which users are situated. In this study, Instagram’s algorithm will be somewhat shaped by the demographic characteristics and user tastes/preferences within the Canadian context specifically.

7. We follow Bishop’s (2025) definition of influencers as ‘ostensibly ordinary platform users who accrue followings on social media via “authentic” (yet stylised) coverage of their everyday lives’ (p. 2110).

8. A definition of what constituted health and fitness content was not provided at the recruitment stage or when communicating the data collection task to participants; instead, participants were encouraged to interpret and define this content based on their own experiences.

9. All interviews were conducted in English. While many participants were bilingual and spoke additional languages (such as Cantonese or Mandarin), English was the first language for many participants, particularly second-generation women.

10. The ‘discovery page’ on Instagram, also known as the Explore page, is a personalized feed that showcases content tailored to a user’s interests, preferences and past interactions. It serves as a curated collection of photos, videos, and other media from accounts that the user may not follow but might find engaging or relevant based on their activity on the platform.
